# 

**Published:** 1781-02

**Authors:** 


### SECTION IV.

### Monthly Catalogue.

1. MODERN improvements in the Practice of Surgery, by Henry Manning, M.D. 8vo. 423 pages, London; 1780.

There is no ſuch perſon as Henry Manning, M.D.—In our laſt monthly catalogue we announced another work under the ſame name, entitled, *Modern Improvements in the Practice of Phyſic.* The two volumes may be conſidered as uſeful compilations from the beſt modern Engliſh medical and ſurgical writers; but they would have been more valuable if the editor had drawn his materials from a greater variety of ſources, and given his readers abſtracts of foreign works of merit that are not ſufficiently known in this country. At the end of the medical volume, we meet with ſome account of the remedies that have been introduced into practice of late years; but what is ſaid on theſe ſubjects is in general not ſufficiently ſatisfactory; the account given of the columbo root, for inſtance, is comprized in half a dozen lines.

2. *C. E. Gellert*, Anfangſgründe zur Metallurgiſchen chymie in einem theoretiſchen und praktifchen Theil. mitkupfern. Zweyte vermehrte und verbefferte Aufgabe. *i. e.* Elements of the Theory and Practice of Metallurgical Chemiftry, by *C. E. Gellert*, with copper-plates, fecond edition, corrected, with additions, 8vo. Leipfic, 498 pages.

The firft edition of this work was publifhed in 1750. In the prefent edition the author, who is infpector of the mines in Saxony, has availed himfelf of all the improvements that have been made in metallurgical chemiftry fince that period. Mr. Gellert informs us, that he has fometimes diffolved copper filings, and likewife thin plates of copper, by mixing them with charcoal, and expofing them, in clofe veffels, to a long continued moderate heat. By means of this procefs he has obtained fometimes a grey, and fometimes a beautiful red *vitrum cupri*, which was increafed $\frac{1}{8}$ part in its weight. In a very ftrong fire this fubftance was again deprived of its phlogifton and a pure malleable copper regenerated. From this circumftance it may be concluded that phlogifton alone is fometimes employed by nature to mineralize copper, and perhaps fome other metals likewife. Our author does not confider the regulus obtained from cobalt as a peculiar femimetal, but as a compofition of iron and arfenic with another fubftance as yet unknown; and he is is of opinion that the blue colour cobalt imparts to glaſs, depends at leaſt in a certain degree on iron.

3. Anfangſgründe zur Probierkunſt, *i. e.* Elements of the Art of aſſaying Metals, by *C. E. Gellert*, a new edition, with additions. 8vo. Leipſic. 176 pages, with copper-plates.

This work, as well as the one laſt mentioned, has received many valuable additions from the author's own experience.

4. *William Cullen*, D. A. D. &c. Anfangſgründe der Praktiſchen Arzneywiſſenſchaft, *i. e.* Firſt Lines of the Practice of Phyſic, by William Cullen, M. D. &c. Parts 1 and 2, 8vo. Leipſic.

5. *Samuel Foart Simmons*, D. A. D. &c. Anatomie des Menſchlichen Körpers, oder Beſchreibung des Baues deſſelben. Aus dem Engliſchen, und mit des überſetzen eigenen Anmerkungen vermehrt und verbeſſert, *i. e.* The Anatomy of the Human Body, or a Deſcription of its Structure, by *Samuel Foart Simmons*, M. D. &c. tranſlated from the Engliſh, with notes by the Tranſlator. 8vo. Leipſic.

6. Obſervations ſur la Rage; ſuivies de Reflexions critiques ſur les Preſervatifs de cette Maladie, par *M. le Roux*, Maitre en Chirurgie, Aſſocié de l'Academie Royale des Sciences de Dijon, et

Chirurgien

Chirurgien Major de l'Hopital General de la meme ville. Dijon, 8vo, 52 pages.

The author of this pamphlet (which, though it contains nothing new, is judiciously written) is very properly of opinion that none of the remedies that have hitherto been recommended with a view to prevent the hydrophobia, are deserving our confidence. He thinks with some other late writers that our principal attention ought to be directed to the wound through which the poison may be expected to enter the habit, and accordingly recommends scarification, topical bleeding, the cautery, &c. in order to prevent an absorption of the virus.

7. Observations rares de medecine, d'anatomie, et de chirurgie, traduites du Latin de Vander Wiel, par *M. Planque*, M. D. avec figures. Paris, 12mo. 2 Vols. containing each about 550 pages.

8. Experiences, et observations sur differentes especes d'air, ouvrage traduit de l'Anglois de *M. Priestley*, Docteur en Droit, membre de la Société Royale de Londres. Par *M. Gibelin*, M. D. Membre de la Société Medicale de Londres. Tomes IV and V. Paris, 8vo.

9. Instruccion curativa de los Tabardillos; par el *Don Josef Amar*, M. D. &c. *i. e.* Remarks on on the cure of eruptive difeafes, by *Don Jofeph Amar*, M. D. Phyfician to his Majefty, Counfellor of the Royal Tribunal of Phyfic, firft Phyfician for the kingdom of Navarre, Member of the Royal Society of Sciences at Seville, and Vice Prefident of the Royal Medical Academy at Madrid. 4to, Madrid.

In this work, which confifts of 327 pages, we read much of putridity and malignancy, of crudity and coction. Lewis de Toro, John de Carmona, and other Spanifh medical writers are the moft frequently quoted. The work clofes with an account of the different epidemics that have prevailed in Spain.

10. Inftruccion curativa y prefervativa de dolores de coftado y pulmonias; *i. e.* Remarks on the cure of difeafes of the breaft and lungs. *By the fame.* 4to. Madrid, 204 pages.

The author divides pleurify into the afcending and defcending, and from thefe diftinctions derives the indications for bleeding or purging in this difeafe.

11. Les grands remedes contre la rage, l'épilepfie, les vertiges et vapeurs qui ont atteints a ce mal, et autres infirmités; par M. *Le Jayant*, Curé de Nôtre Dame de la Quinte près de Mans. 8vo. Mans.

This

This Curate of La Quinte may not improperly be ranked as a medical writer with our country-man Mr. Wefley.

12. Hiftoire Medicale des Maladies dyfenteriques qui affligerent la province de Maine en 1779; moyens convenables pour combattre avec fuccés le mal principal et les accidens qui en font la fuite; par M. *Vetillard*, M. D. Membre du College des Medecins de Mans. 12mo. Mans. 74 pages.

This feems to be the production of an experienced and judicious practitioner.

13. De China China in Synochis Putribus animadverfiones *Petri Joannis Vaftpania* Amplisfimi Taurinenfis Medicorum Collegii Socii, nec non Medici in Nofocomio urbis Majori Vicarii. Taurinii, 8vo, 152 pages.

The author of this Work condemns the Peruvian bark, but his arguments are by no means well founded.

14. Le Citoyen Dentifte, ou l'art de feconder la Nature pour fe conferver les dents, et les entretenir propres. Ouvrage Moderne, a la portée de tout le Monde. Par *M. Hubert*, Chirurgien dentifte, recû au College Royal de Chirurgie de Paris, dentifte penfionné de la Ville. 12mo. Lyons, 95 pages.

